# Therapeutic optimization through goal-oriented prescription in nursing homes

**DOI:** 10.1007/s11096-020-01206-x

**Published:** 2020-11-28

**Authors:** N. Molist-Brunet, D. Sevilla-Sánchez, J. González-Bueno, V. Garcia-Sánchez, L. A. Segura-Martín, C. Codina-Jané, J. Espaulella-Panicot

**Affiliations:** 1 Hospital Universitari de la Santa Creu de Vic, Vic, Barcelona Spain; 2 Central Catalonia Chronicity Research Group (C3RG), Centre for Health and Social Care Research (CESS), Universitat de Vic—University of Vic-Central University of Catalonia (UVIC-UCC), C. Miquel Martí i Pol, 1, 08500 Vic, Spain; 3 Pharmacy Department, Hospital Universitari de Vic, Vic, Barcelona Spain; 4 Pharmacy Area, Institut Català de la Salut, Barcelona, Spain; 5 Institut Català de la Salut (ICS), Barcelona, Spain; 6 Geriatric and Palliative Care Department, Hospital Universitari de Vic, Vic, Barcelona Spain

**Keywords:** Goal-oriented prescription, Inappropriate prescription, Nursing homes, Polypharmacy

## Abstract

*Background* People living in nursing homes are highly vulnerable and frail. Polypharmacy and inappropriate prescription (IP) are also common problems. *Objectives* The objectives of the study are (i) to study the baseline situation and calculate the frailty index (FI) of the residents, (ii) to assess the results of routine clinical practice to do a pharmacotherapy review (patient-centred prescription (PCP) model) (Molist Brunet et al., Eur Geriatr Med. 2015;6:565–9) and (iii) to study the relationship between IP and frailty, functional dependence, advanced dementia and end-of-life situation. *Setting* Two nursing homes in the same geographical area in Catalonia (Spain). *Method* This was a prospective, descriptive and observational study of elderly nursing home residents. Each patient’s treatment was analysed by applying the PCP model, which centres therapeutic decisions on the patient’s global assessment and individual therapeutic goal. *Main outcome measure* Prevalence of polypharmacy and IP. *Results* 103 patients were included. They were characterized by high multimorbidity and frailty. Up to 59.2% were totally dependent. At least one IP was identified in 92.2% of residents. Prior to the pharmacological review, the mean number of chronic medications prescribed per resident was 6.63 (SD 2.93) and after this review it was 4.97 (SD 2.88). Polypharmacy decreased from 72.55% to 52.94% and excessive polypharmacy fell from 18.62% to 5.88%.The highest prevalence of IP was detected in people with a higher FI, in those identified as end-of-life, and also in more highly dependent residents (*p* < 0.05). *Conclusions* People who live in nursing homes have an advanced frailty. Establishing individualized therapeutic objectives with the application of the PCP model enabled to detect 92.2% of IP. People who are frailer, are functionally more dependent and those who are end-of-life are prescribed with inappropriate medication more frequently.

## Impacts on practice

- This study shows how the application of a pragmatic framework enables individualizing prescriptions for frail patients in their daily care.
- It also demonstrates the ability of the proposed framework to optimize prescriptions and to reduce polypharmacy in this patient profile.

## Introduction

People who live in nursing homes share a high vulnerability and frailty [1]. They are usually characterized by high functional dependence. Taking as a reference different studies in UK, we consider that overall, 76% of residents require assistance with their mobility or are immobile, while 78% have at least one form of mental impairment and 71% are incontinent [2].

Frailty [3] (common clinical syndrome in elderly that carries an increased risk of poor health outcomes) is identified in almost all nursing home residents, and in most cases, it is moderate or severe [4]. Frail people most often experience falls, immobility and confusion syndromes, as outlined in a review of nursing homes in the UK [4].

Additionally, also in UK nursing homes, a large number of residents are end-of-life. Mortality at six months after entering a nursing home is around 16% and at twelve months approximately 25% [5].

The situation in nursing homes in Catalonia appears to be similar: residents are highly functionally dependent (90% require mobility assistance) and 55–60% suffer at least one form of mental impairment. Mortality at twelve months is around 15.4%) [6, 7]

Polypharmacy and inappropriate prescriptions (IP) are common problems in nursing homes [8]. Polypharmacy has proved to be a risk factor for negative health outcomes, in addition to involving major healthcare expenditure [9]. IP is more common in people with multimorbidity and it accounts for 30% of emergency hospital admissions from nursing homes [8]. Previous studies have demonstrated increased mortality in people taking six or more medications, with a greater risk if they take 10 or more [9].

The strict application of clinical practice guidelines in people with multimorbidity leads to polypharmacy, and it does not provide guidance on how best to prioritize recommendations for individuals [10]. Given the marked vulnerability of nursing home residents, there is concern and evidence that they may not benefit from aggressive management of chronic conditions in the same way that study populations do. Therapy for hypertension or dyslipidaemia in nursing homes are examples of the risk of overtreatment [11]. Therefore, it becomes essential to ensure that the benefit of treatment outweighs the harm in very vulnerable residents, in whom the risk of side effects may be particularly high [11, 12].

Consequently, it is imperative to carry out a periodic review of pharmacological prescription in nursing homes. Interdisciplinary work by geriatricians and clinical pharmacists has demonstrated the ability to reduce polymedication, identify IP and prevent potential adverse events related to medication [13].

However, there are still doubts concerning the benefits that medication review can bring with regard to reducing hospital admissions, improving quality of life or decreasing mortality [13, 14].

In this regard, according to the Chronicity Plan of Catalonia, a prescription based on individualized therapeutic goals is endorsed for clinical practice [15].

**Fig. 1:**
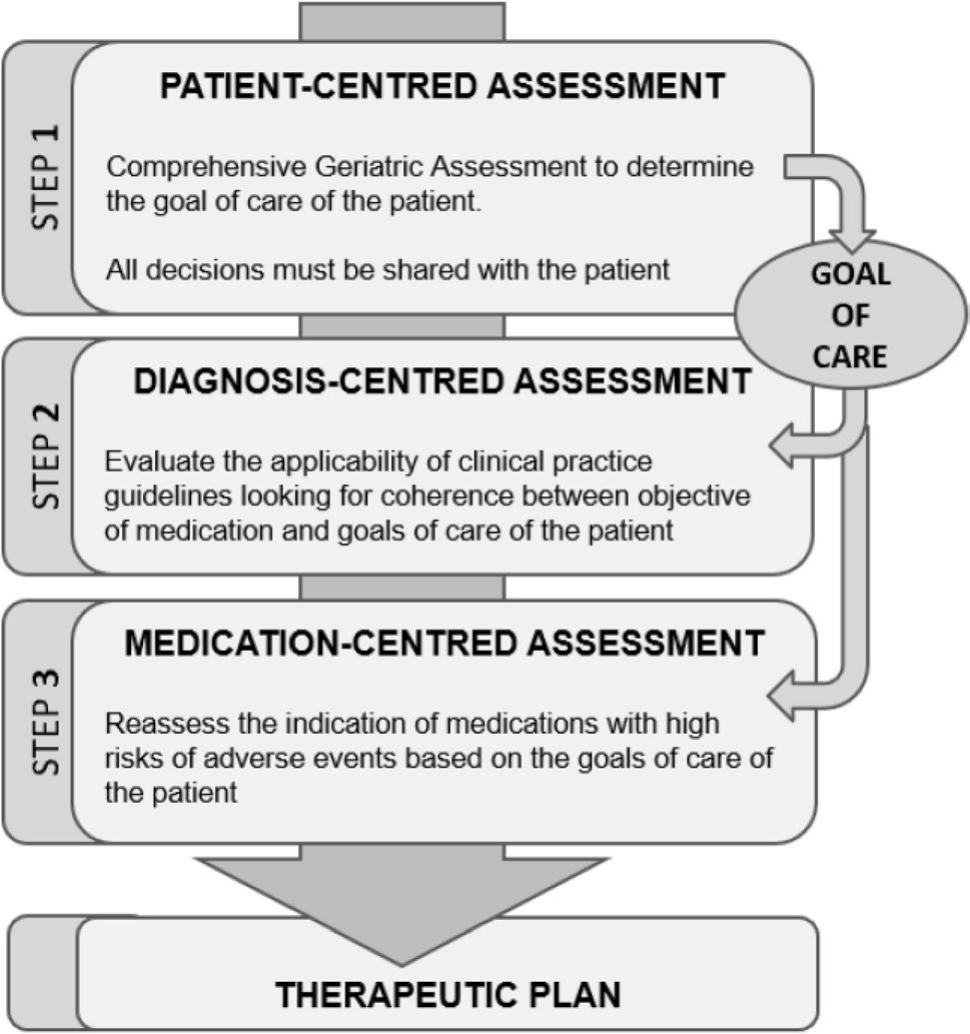
Patient-centred prescription (PCP) model

*Pharmacotherapy review* Each patient’s treatment was analysed by applying the patient-centred presciption (PCP) model [16]. This is a systematic 4-stage process, carried out by a multi-disciplinary team formed by a geriatrician, a nurse and a clinical pharmacist. The model centres therapeutic decisions on the patient’s global assessment (comprehensive geriatric assessment (CGA), calculation of the frailty index (Frail-VIG) [17]) and the resulting individual therapeutic goal (prolonging survival, maintaining functionality or prioritising symptomatic control) [18]. The decisions were taken in conjunction with the patient or with the main carer in cases of incapacity (Fig. 1)

Different criteria were used to determine IP: (i) Residents at the end of life (according to NECPAL CCOMS-ICO© [19]): the indication of medications aimed at prolonging survival was reassessed. Medications for primary prevention were evaluated for potential discontinuation and those for secondary prevention were individualized in accordance with patient goals [18]. (ii) Type 2 Diabetes Mellitus (T2DM): to optimize hypoglycaemic therapy two important proposals were considered: *Therapeutic intensity criteria* (taking American Diabetes Association (ADA) guidelines as our basis) [20–22], we established a maximum HbA1c target for each patient profile, determined by the therapeutic goal agreed on by applying the PCP model. In accordance with the HbA1c target, therapeutic modifications were proposed: an increase or decrease in dose or the start or withdrawal of treatment, according to each case (Table 1). And *qualitative criteria regarding drug prescription* to consider IP (the prescription of sulphonylureas (SU) was considered inappropriate due to their high risk of hypoglycaemia [21, 23]; patients with doses of metformin not adjusted for renal failure [21]; patients with gliflozins (SGLT2 inhibitors) and renal failure (glomerular filtration rate (GFR) < 45 ml/min) [21, 24] and, the use of insulins associated with the highest risk of hypoglycaemic episodes (short-acting insulins, mixtures and postprandial use) was considered inappropriate, except in justified cases [21]). (iii) Hypertension and cardiovascular therapy: There is little evidence regarding the specific objectives of blood pressure levels in elderly and frail people. There are currently several pieces of evidence that recommend less intensive control in people with multimorbidity, especially in cases of dementia or limited life expectancy [25]. In general, blood pressure lower than 140/90 mmHg has been associated with a higher risk of falls and even mortality [8, 26, 27]. In our study we have proposed measures for pharmacological adjustment in people whose mean systolic blood pressure (SBP) was under 130 mmHg in the last year. (iv) Dyslipidaemia: statins are not recommended for primary prevention in end-of-life patients, regardless of the indication. In the case of secondary prevention, we individualized decision-making based on the associated risks and benefits for each patient [18]. Withdrawal of lipid-lowering medication was suggested for people who had total cholesterol (TC) lower than 150 mg/dl, given that it is a malnutrition marker [28]. (v) Mental health and Dementia: the recommendations of the European Association of Palliative Care were followed. They define a different therapeutic objective in patients with dementia according to the evolutionary stage of their pathology. It is based on evidence and consensus among experts [29]. Regarding chronic antipsychotic treatment, the progressive decrease in doses was proposed in people who had not had behavioural disorders in last 3–6 months [30, 31]. (vi) Pain: in accordance with Beers/STOPP criteria, the following proposals were made [32]: Tricyclic antidepressants to treat neuropathic pain were avoided, due to their anticholinergic effects; non-steroidal anti-inflammatory drugs (NSAID) were recommended at the lowest dose and for the shortest time possible, due to the high incidence of adverse drug events (ADE) and weak opioids such as tramadol and codeine were recommended only at low doses due to the risk of ADE. When higher doses were needed, a change to morphine treatment was proposed to avoid anticholinergic effects. (vii) Osteoporosis: the withdrawal of supplementary treatments with calcium, vitamin D or bisphosphonate was suggested for people who were not mobile [31].

**Table 1:** Therapeutic goals in Type 2 Diabetes Mellitus (T2DM) according to patient profile

Patient profile	Qualitative glycaemic target	Quantitative glycaemic (HbA1c) target	Therapeutic goal proposed by PCP^a^ model
Healthy elderly patients with good functional and cognitive status, a low burden of comorbidities and a long life expectancy	Targets can be similar to those for young adults with diabetes	≤ 7.5%	To prolong survival
Frail elderly patients with functional disability, dementia or moderately limited life expectancy	Symptomatic hypoglycaemic episodes and symptomatic hyperglycaemia should be particularly avoided	≤ 8.0%	To maintain functionality
Elderly patients in a probable end-of-life situation, understood as a period of 1–2 years	The priority should be to preserve the quality of life, avoiding symptomatic hypoglycaemic episodes and symptomatic hyperglycaemia	≤ 8.5%	To give priority to symptomatic treatment

^a^PCP Model: Patient-centred prescription model

### Aim of the study

- To study the baseline situation and calculate the frailty index (FI) of the nursing home residents.
- To assess the results of routine clinical practice to do a pharmacotherapy review (PCP model) [16]:
  - Polypharmacy prevalence.
  - To identify IPs and optimize them.
- To study the relationship between IP and frailty, functional dependence, advanced dementia and end-of-life situations.

### Ethics approval

The study was based on the collection of data generated in the clinical practice. Thus, informed consent was not considered necessary since inclusion in this study did not constitute undergoing a specific intervention. Additionally, we obtain the verbal informed consent from the patient or the main caregiver. Afterwards, we include the patient’s verbal informed consent in their electronic health record. The study was approved by the local Scientific Ethics Committee of the Hospital Universitari de Vic, under reference number 2019-106/PR237.

## Method

### Design

This was a prospective, descriptive, observational study of elderly care home residents from two nursing homes in the same geographical area in Catalonia (Spain). Data were collected from February to July 2019.

*Data collected* (i) Personal data (age and gender). (ii) Functional data (dependence/independence for instrumental activities and the Barthel Index (BI) to assess basic activities of daily living [33]). (iii) Medical data (total number of comorbidities; dementia diagnosis, as stated in the medical records, and the degree of deterioration was established in accordance with GDS (Global Deterioration Scale) for Alzheimer-type dementia and with Clinical Dementia Rating (CDR) for the rest [34, 35]; blood pressure levels available in the last year). (iv) Analytical data (full blood count; ionogram; urea; electrolytes and glycosylated haemoglobin (HbA1c) available in the last year). (v) Pharmacological data (total number of chronic drugs taken by each resident (for at least six months) in baseline and post-review; polypharmacy (5 or more medications) or excessive polypharmacy (10 or more medications) [36] in baseline and post-review; and total number of high iatrogenic risk medications (insulin and oral hypoglycaemic agents (except metformin), antithrombotic drugs, opioids, NSAIDs, digoxin and anti-psychotic agents) [37, 38]). (vi) Frailty (measured by the Frail-VIG index (FI)) [39, 40] and categorized (as FI < 0.20: no frailty; FI 0.20–0.35: mild frailty; FI 0.36–0.50: moderate frailty and FI > 0.50: severe frailty).

*Identification of end-of-life patients (EOL patients)* (using NECPAL CCOMS-ICO© tool criteria) [19]: these are patients considered to be in the final months or year of their life. The criteria used to identify them as EOL patients were: (i) identification as such by their primary care physician, (ii) advanced illness criteria [19] or (iii) Frail-VIG index >0.50.

*Inclusion criteria* Patients older than seventy living in nursing homes

*Exclusion criteria* Diagnosis of a major mental disorder, such as schizophrenia or mental handicap, and residents in their probable last hours or days of life [41].

### Sample size

To calculate sample size, IP in the overall elderly nursing home population was estimated at 80% [8]. With a 95% confidence level and 8% accuracy, 87 residents should be included. However, data collection was prolonged to complete the calendar month, despite the fact that this involved an increase in the previously calculated size.

### Statistical methods

Statistical analysis was performed with IBM SPSS Statistics v23.0 statistical software. The results for categorical variables were expressed as absolute and relative frequencies and results for continuous variables were analysed using both parametric and non-parametric statistics, depending on the level and distribution of data (as means and SD or median, 25 and 75 percentiles and minimum and maximum values). The statistical tests used to evaluate the relationship between two qualitative variables were the Chi-square test (with Yates’ correction if necessary, or Fisher’s exact test in 2 × 2 tables where the expected frequencies were lower than 5). Student’s t-test was used to analyse the relationship between quantitative and qualitative variables for the variables that followed the normal distribution, or the Mann–Whitney U for those variables that did not follow it. Statistical significance was considered when the value of *p* was less than 0.05.

## Results

Of a total of 160 nursing home residents, 103, of whom 69.9% (*n* = 72) were women, met the inclusion criteria, with an average age of 83.1 years (SD 8.72).

The residents in the sample were characterized by high dependence in their baseline situation: 100% were dependent for instrumental activities and the median BI was 20/100 (P25: 10; P75: 50). Up to 59.2% (*n* = 61) were totally dependent (BI under 20). 88.3% (*n* = 91) of residents had some kind of incontinence and 55.3% (*n* = 57) were double incontinent. Up to 79.6% (*n* = 82) had a diagnosis of dementia which in 64.1% (*n* = 66) of cases was moderate or severe. Regarding their emotional assessment, 52.4% (*n* = 54) had a diagnosis of depressive syndrome and received specific treatment. 63.1% (*n* = 65) of those with affective disorders took specific medication.

On average, they had five comorbidities (minimum one and maximum nine). Hypertension (60.2%) (*n* = 62), T2DM (28.1%) (*n* = 29), chronic kidney failure (CKF) (24.3%) (*n* = 25), heart failure (22.3%) (*n* = 23) and dyslipidaemia (20.4%) (*n* = 21) were the most frequent.

Most residents had moderate or severe frailty (81.6%) (*n* = 84), and in particular 26.47% were severely frailty. The median FI was 0.44 (minimum 0.20 and maximum 0.72) (Table 2).

**Table 2:** Frailty prevalence according to the VIG-Frail index

VIG-Frail index	Number of people (%)
IF < 0.20: no frailty	0 (0%)
IF 0.20–0.35: mild frailty	19 (18.4%)
IF 0.36–0.50: moderate frailty	55 (53.4%)
IF > 0.50: severe frailty	29 (28.2%)

According to NECPAL CCOMS-ICO© criteria [19], 53.4% (*n* = 55) of residents were classified as being end-of-life and were identified as NECPAL positive. NECPAL positive residents had higher FI, and presented moderate frailty (47.3%) (*n* = 26) or severe frailty (50.9%) (*n* = 28) in 98.2% of cases.

An individual therapeutic goal was established for each resident according to their baseline situation. The most frequently established therapeutic goal was to maintain functionality (50.4% (*n* = 52)); in second place came symptomatic control prioritization (46.6% (*n* = 48)). Only in 2.91% (*n* = 3) cases was survival prioritization the main therapeutic goal.

Prior to the pharmacotherapy review, the mean of chronic prescription medications per resident was 6.63 (SD 2.93). Of these, 25.3% were for preventive purposes, 41.8% etiological and 32.9% symptomatic. Up to 83.4% of residents took at least one high iatrogenic risk medication (antithrombotic, hypoglycaemic agents, antipsychotic medications, diuretics, NSAIDs and digoxin) [37, 38, 42].

At least one IP was identified in 92.2% of home residents (*n* = 95). The most frequent categories of IP were alimentary tract and metabolism (30.05%), cardiovascular system (25.39%) and nervous system (24.87%) (Table 3).

**Table 3:** Inappropriate prescriptions identified according to the ATC (Anatomical, Therapeutic, Chemical classification) system

ATC group	Total
A-Alimentary tract and metabolism	58 (30.05%)
B-Blood and blood-forming organs	17 (8.80%)
C-Cardiovascular system	49 (25.39%)
D-Dermatological	0
G-Genito-urinary system and hormones	5 (2.59%)
H-Systemic hormonal preparations, excluding sex hormones and insulins	0
J-Antiinfectives for systemic use	2 (1.04%)
L-Antineoplastic and immunomodulating agents	0
M-Musculoskeletal system	7 (3.63%)
N-Nervous system	48 (24.87%)
R-Respiratory system	5 (2.59%)
S-Sensory organs	2 (1.04%)
V-Various	0

In total, 273 proposals for therapeutic modifications were made, resulting in a mean of 2.65 (SD 1.59) proposals per resident assessed. Of these proposals, 212 were able to be implemented (77.65%), with a mean of 2.06 (SD 1.33) per resident assessed. The remaining 61 proposals were deferred to the next follow-up visit, with the aim of introducing the changes progressively.

**Fig. 2:**
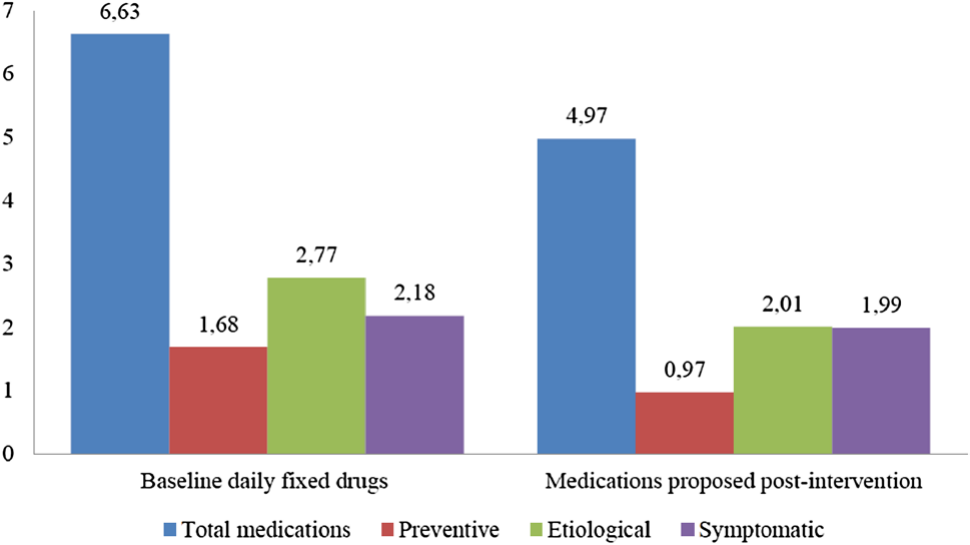
Comparison between baseline daily fixed drugs and those proposed post-review

**Fig. 3:**
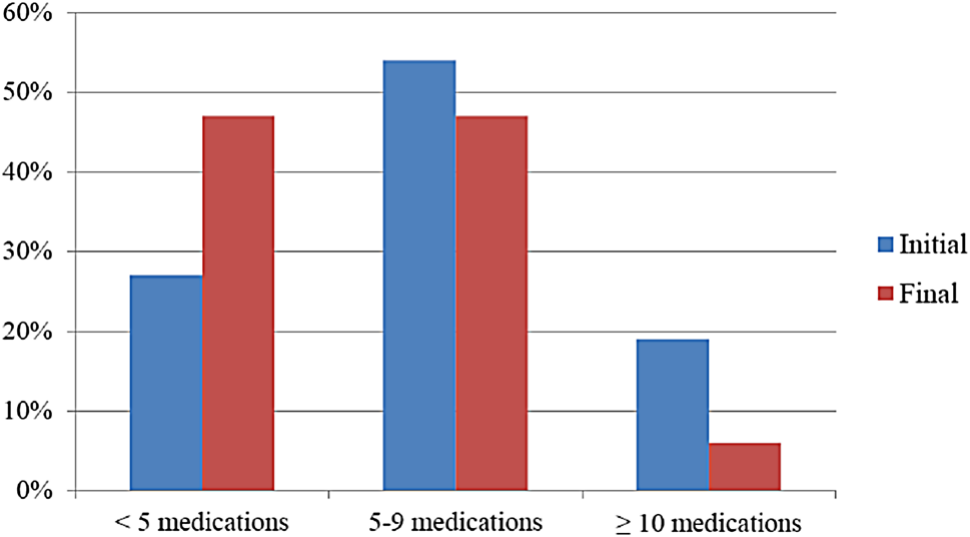
Comparison of polypharmacy in baseline prescription and post-review

After the application of the PCP model, residents took an average of 4.97 (SD 2.88) chronic medications (19.5% for preventive purposes, 40.4% etiological and 40.1% symptomatic) (Fig. 2). As a final result, the number of chronic medications proposed decreased by 25.15%. Polypharmacy decreased from 72.55% to 52.94% and excessive polypharmacy from 18.62% to 5.88% (Fig. 3).

The highest prevalence of IP was detected in people with higher FI, those identified as end-of-life and also in people with higher dependence (*p* < 0.05) (Table 4). Relevant clinical differences were also observed in people with advanced dementia who had a higher IP but were not statistically significant (*p* > 0.05)

**Table 4:** Relationship between IP and FI, functional dependence and identification of end-of-life situation

		Inappropriate prescription		*p*-value
		YES 95 (92.2%)	NO 8 (7.8%)	
NECPAL Positive				
YES		54 (98.2%)	1 (1.8%)	**0.024**
NO		41 (85.4%)	7 (14.6%)	
Advanced dementia				
YES		41 (95.3%)	2 (4.7%)	>0.05
NO		54 (90.0%)	6 (10%)	
Frailty index (FI), mean (SD)		0.43 ± 0.09	0.36 ± 0.11	**0.043**
Functional dependence (BI), median (P25–P75)		20 (10–45)	70 (28.8–85)	**0.010**

The bolded values are used to highlight when *p* value is significant

## Discussion

The study describes a sample of institutionalized people from a specific county with a similar profile to that described in previous studies that focus on pharmacotherapeutic review: very old people, a predominance of women and a high proportion of people with significant dependence, multimorbidity and cognitive impairment [1].

A prevalence of incontinence greater than that identified in the literature was detected (88.2% compared to a maximum of 71% in the literature) [2, 43]. The prevalence of depressive syndrome with a specific treatment was also higher than that reported in other studies (51.96% compared to 37.5%) [44]. Diagnosis of dementia was similar to that found in the literature (80.31% compared to 78%) [2]. In contrast, the proportion of patients with cognitive impairment who had prescribed fixed antipsychotic treatment was much higher in our study (49.01% compared to 25% [45]).

Regarding baseline pharmacological data, the average of chronic medications was lower than usual in institutionalized patients, which is usually around nine. This is probably due to the fact that a review of the medication had been carried out in these two nursing homes two years before, in conjunction with a clinical pharmacist.

It is remarkable that in a subgroup of particularly vulnerable patients, more than 80% are exposed to at least one drug with a high iatrogenic risk.

The number of patients with at least one IP (92.2%)—similar to that detected with the application of STOPP–START and Beers criteria [8] in other studies—reveals a great need to optimize prescription for institutionalized people. On the other hand, it is important to point out, just as other studies have highlighted, that the analysis of eight pharmacological groups enables detecting over 80% of IP.

The PCP methodology enabled the immediate implementation of a high proportion of the therapeutic proposals (77.65%). In addition, in nursing homes it is particularly feasible to apply the rest of the proposals progressively and to keep track of the changes. Although the number of baseline medications was not particularly high, the application of the PCP model led to the removal of a quarter of chronic medications. The prescription of new medications is usually based on the recommendations of the clinical practice guidelines, which usually pursue a survival objective. However, if the individual goal is reassessed, the prioritization of survival is indicated in only a small proportion of the residents. Consequently, the therapeutic plan is greatly modified, both quantitatively (diminished polypharmacy) and qualitatively (with a decrease in the proportion of preventive medications and a relative increase in symptomatic medications).

This work confirms that the frailer and the nearer the patient is to the end of their life, the greater the probability of suffering IP increases. Therefore, care based on the therapeutic objective is a highly important work methodology since it facilitates individualized intervention, especially in the most vulnerable situations. The PCP model is a tool that clearly facilitates this type of intervention. Thus, the goal-based methodology enables decisions to be taken beyond the scope of medications, opening the door to facilitate decisions on therapeutic intensity in future crises.

The methodology developed with the PCP model demonstrates the great importance of interdisciplinary work, in this case between nurses, the physician and the clinical pharmacist.

Nursing homes concentrate a large proportion of the most vulnerable people in the community; this could make these centres benchmarks for care for multimorbidity patients and end-of-life chronic diseases. This could have an impact not only on residents’ health outcomes, but also on the hospital environment, especially regarding emergency services, where patient numbers could be reduced.

The current study has some limitations. Firstly, we performed an analysis of the medications for the listed chronic conditions but a calculation of anticholinergic burden, which is also very important in people living in nursing homes, is lacking.

Secondly, we reviewed only 103 of the 160 residents’ pharmacological therapeutic plans. We excluded younger residents and the ones whose principal diagnosis was a major mental disorder due to the team’s geriatrics approach.

In addition, we included a few more patients than our calculation recommended due to the large differences existing in the literature regarding IP prevalence.

Finally, these results enable us to confirm that the application of the PCP model in clinical practice considerably optimizes pharmacological prescription in frail nursing home patients.

## Conclusions

People who live in nursing homes have a high prevalence of morbidity, advanced frailty and cognitive impairment. In fact, more than half of the institutionalized population are possibly at the end of life.

Establishing an individualized therapeutic objective with the application of the PCP model resulted in the detection of 92.2% IP in a nursing home. Consequently, polypharmacy decreased from 72.55% to 52.94% and excessive polypharmacy from 18.62% to 5.88%.

People with greater frailty, greater functional dependence and those who are end-of-life are prescribed with inappropriate medication more frequently.
